# Effect of a Real-Time Audio Ventilation Feedback Device on the Survival Rate and Outcomes of Patients with Out-of-Hospital Cardiac Arrest: A Prospective Randomized Controlled Study

**DOI:** 10.3390/jcm12186023

**Published:** 2023-09-18

**Authors:** Eun Dong Lee, Yun Deok Jang, Ji Hun Kang, Yong Song Seo, Yoo Sang Yoon, Yang Weon Kim, Woong Bin Jeong, Jae Gu Ji

**Affiliations:** Department of Emergency Medicine, Inje University Busan Paik Hospital, 75, Bokji-ro, Busanjin-gu, Busan 47392, Republic of Korea; led3009@naver.com (E.D.L.); jangyundeok@naver.com (Y.D.J.); mona@hanmail.net (J.H.K.); seoyongsong@gmail.com (Y.S.S.); 101mars@hanmail.net (Y.S.Y.); ywked@inje.ac.kr (Y.W.K.); emt_jwb@naver.com (W.B.J.)

**Keywords:** out-of-hospital cardiac arrest, advanced cardiac life support, audio ventilation feedback, cerebral performance category, emergency medical department

## Abstract

The purpose of this study was to evaluate the effect of real-time audio ventilation feedback on the survival of patients with an out-of-hospital cardiac arrest (OHCA) during advanced cardiac life support (ACLS) performed by paramedics. This research was a prospective randomized controlled study performed in Busan, South Korea, from July 2022 to December 2022. This study included 121 patients, ages 19 and up, who were transferred to the study site, excluding 91 patients who did not receive CPR under a doctor’s direction as well as those who had a ’(DNR)’ order among 212 adult CA patients. OHCA patients’ clinical prognosis was compared by being randomly assigned to either a general manual defibrillator (NVF) group (N = 58) or a manual defibrillator with an audio ventilation feedback (AVF) group (N = 63). To verify the primary outcome, the cerebral performance category (CPC), return of spontaneous consciousness (ROSC), 30h survival, and survival discharge were compared. Multivariate logistic regression was conducted to analyze the association between the audio-feedback manual defibrillator (AVF) and the ROSC of OHCA patients. This study analyzed 121 patients among 212 OHCA patients. The ROSC (AVF group: 32 {26.4%} vs. NVF group: 21 {17.3%}), 24 h survival (AVF group: 24 {19.8%} vs. NVF group: 11 {9.0%}), and survival discharge (AVF group: 12 {9.9%} vs. NVF group: 6 {4.9%}) were higher in the AVF group than the NVF group. However, upon analyzing CPC scores in the surviving patients between the two groups, there was no significant difference (AVF group: 4.1 ± 1.23 vs. NVF group:4.7 ± 1.23, *p* = 1.232). Multivariate logistic regression analysis showed that the use of AVF was associated with a higher ROSC (odds ratio {OR}, 0.46; 95% confidence interval {CI}, 0.23–0.73; *p* < 0.01) and higher survival at 30 h (OR, 0.63; 95% CI, 0.41–0.98; *p* = 0.01).

## 1. Introduction

High-quality CPR is a key clinical procedure performed on patients with cardiac arrest (CA) [1] because it has an important effect on the resuscitation of cardiac arrest patients and has a good outcome on neurological prognosis [2,3]. CPR provided to CA patients consists of chest compression and artificial respiration. The number of chest compressions and the method of respiration may differ depending on whether or not a professional advanced airway device is inserted [4]. The recommendation of the American Heart Association is that, in situations where specialized instrumental intubation has not been performed, 30 chest compressions at a depth of 5 cm at the center of the sternum and subsequent complete relaxation should be achieved at a rate of 100 to 120 compression per minute [4]. In situations where professional airway-securing surgery has not been performed, 30 chest compressions should be completed, and artificial ventilation should then be performed twice per second [5,6]. Alternatively, in patients who have undergone professional airway acquisition, chest compression should be performed for two minutes. Back valve mask ventilation is performed once every six seconds, but optimal ventilation is recommended while squeezing only one-third of the bag [7]. According to previous studies, if hyperventilation occurs during cardiopulmonary resuscitation, intrathoracic pressure (ITP) increases and may interfere with cardiopulmonary resuscitation [8,9]. If ITP increases, the first reason it could interfere with CPR is that venous return is not carried out properly, such that the cerebral circulation may be decreased. A secondary reason is that chest compressions become less effective as a result of the pressure [9]. A tertiary reason is that cerebrovascular contraction occurs due to hyperventilation; therefore, many reports indicate that blood flow in the brain may decrease due to a decrease in carbon dioxide partial pressure in the blood [10]. For these reasons, high-quality cardiopulmonary resuscitation is often not provided and may interfere with spontaneous circulation recovery in patients with cardiac arrest. In addition, it does not provide appropriate intracerebral circulation, which adversely affects neurological prognosis. Nevertheless, hyperventilation occurs frequently during CPR by medical providers in hospitals or emergency medical providers outside hospitals for various reasons [11]. Therefore, studies have been conducted to overcome hyperventilation. Some studies have suggested a method of providing accurate artificial respiration speed using metronomes while providing respirations through intratracheal intubation, and studies have been conducted on mannequins to provide ventilation [12]. Another recent study has demonstrated that the use of an impedance threshold device (ITD) helps improve hemodynamic conditions and short-term survival rates in patients with cardiac arrest [13,14,15,16]. And it is helpful that higher regional cerebral oxygenation during resuscitation is associated with an improved return of spontaneous circulation, survival, and neurologic outcomes at hospital discharge [16]. However, long-term survival rates have dropped due to the use of ITDs, and it has been reported that these failures increase intravenous pressure and reduce brain perfusion pressure. It was also reported that using an ITD could produce epileptic pleural fluid and increase pressure in the left ventricle, resulting in heart failure [16]. For these reasons, real-time ventilation feedback sensors have recently been developed to compensate for the shortcomings of ITDs, and equipment that feeds back ventilation speed and ventilation volume in an audio manner has been developed and is in use. However, no studies have been conducted on the effect of these devices on survival rate and prognosis by preventing hyperventilation and providing an appropriate amount of ventilation in high-quality CPR.

Therefore, this author aims to elucidate the effect of the ventilation-provided feedback devices that provide feedback in real time through audio methods that have recently been commercialized on the survival rate and prognosis of cardiac arrest patients.

## 2. Materials and Methods

### 2.1. Study Design and Setting

**Figure 1:**
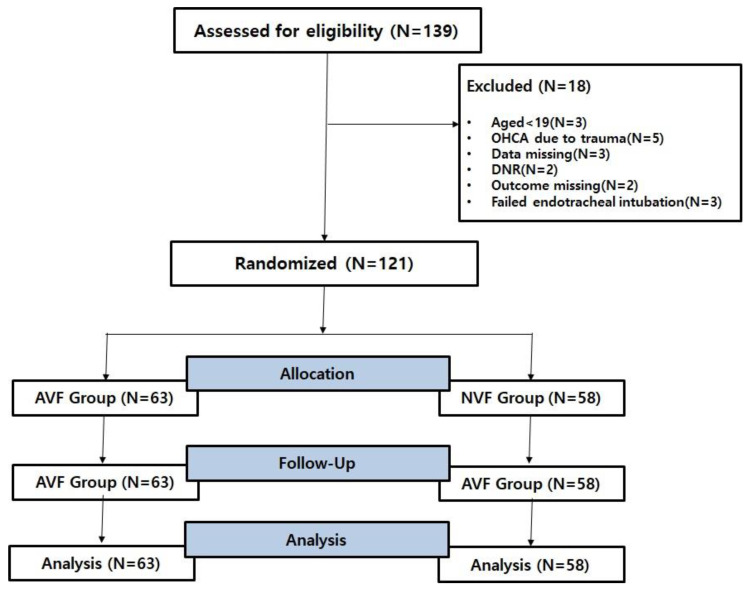
Flow chart of this RCT. RCT, randomized controlled trial; OHCA, out-of-hospital cardiac arrest; DNR, do not resuscitate; AVF, audio ventilation feedback; NVF, non-ventilation feedback. We obtained an RCT number for reference (NIH clinical trial registration number: NCT06903691).

This study was conducted through a prospective, randomized, controlled, single-center design. The patients analyzed amounted to 121 out of 139 who were over the age of 19, were admitted to the ED between 1 July and 1 December 2022, and provided written consent (themselves or via their legal guardians). The research institute that conducted this study is a regional emergency medical center located in Busan, South Korea, where an average of 1000 cardiac arrest patients visit the hospital annually. This study was approved by the Institutional Review Board of Inje University Busan Paik Hospital (IRB approval number: 2021-11-063). The requirement for informed consent was waived due to the anonymous nature of the data. Data on patients with prehospital cardiac arrest were collected with the cooperation of three emergency medical service (EMS) centers near the research institute. The paramedics working at these EMS centers have more than two years of clinical experience and have completed professional cardiac resuscitation and advanced airway management training. Participants were recruited from among on-duty basic life support (BLS) and advanced life support (ALS) providers. All participants were experienced in the use of real-time feedback device on chest compressions during OHCA, but they had no previous experience with the AccuVent sensor or ventilation feedback. Participants were required to be trained BLS or ALS providers who performed CPR as a part of their job description. Furthermore, participants confirmed that they had encountered at least one OHCA within the last 6 months or had attended CPR training within the calendar year. Active first aid instructors, individuals with physical limitations, and those with a known pregnancy were excluded. Between 11 July 2022 and 1 December 2022, CA patients were randomized according to the days on which each method would be used. Randomization was conducted using SAS ver. 9.2 (SAS Institute Inc., Cary, NC, USA). Two defibrillators were used in the study: a defibrillator combined with a device providing audio ventilation feedback (AVF) and a non-ventilation feedback (NVF) defibrillator that was not combined with a device. A flow chart of the study is shown in Figure 1. This study was performed in accordance with the 2021 CONSORT (Consolidated Standards of Reporting Trials) statement and checklist (Figure 1).

### 2.2. Selection of Participants and Data Collection

Data were collected from cardiac arrest patients who were transferred to the emergency medical center, and were among those who received specialized cardiac resuscitation performed by paramedics. The subjects included in the study were patients with cardiac arrest outside the hospital aged 19 and up and were transferred from three EMS centers near the research institution from July 2022 to 1 December 2022. Among them, patients with endotracheal intubation performed by paramedics outside the hospital were selected. If they were physically unable to give consent, a member of their immediate family was asked to sign the consent form on their behalf. During this study, patients under the age of 19, with cardiac arrest due to trauma, with missing CA data, with a DNR order, with missing outcome data, and with failed endotracheal intubation were excluded. The general characteristics of the patients were collected through medical records, and data recorded in the defibrillator’s own database were collected for cardiopulmonary resuscitation-related variables recorded during professional CPR. Variables related to the prognosis of cardiac arrest patients, such as the return of spontaneous consistency (ROSC), death, 80-day survival, and cerebral performance category (CPC), were collected form medical record sheets.

### 2.3. Sample Size Calculation

A total sample size of 139 was calculated using G-Power version 3.1.9.2 (Franz Faul, Univesitat Kiel, Germany) with an alpha probability of 0.05, power of 0.95, and effect size of 0.476. Considering possible dropouts, we decided to include at least 121 patients in this study.

### 2.4. Outcomes

The primary outcome was comparison of survival rates between AVF group and NVF group. And the secondary outcome is the effect on the prognosis of cardiac arrest patients with real-time audio-ventilation feedback.

### 2.5. Data Analysis

Categorical variables are presented as frequencies and percentages, while continuous variables are presented as medians and interquartile ranges (IR). After the normality test, parametric (independent *t*-test or chi-square test) or non-parametric (Mann–Whitney U test) methods were used for comparisons. All statistical analyses were performed using SPSS Statistics version 18.0 (SPSS Inc., Chicago, IL, USA). *p* < 0.05 was considered statistically significant. The main outcome of this study is to find out the survival rate comparison and factors that affect the survival rate after specialized cardiac resuscitation with AVF and NVF. To confirm the primary outcome of this study, a chi-square test was performed. If the continuous variable was normally distributed, a *t*-test test was performed and expressed as the mean and standard deviation. If the normal distribution was not followed, rank sum analysis was performed and expressed as intermediate values and quartiles. To confirm the secondary outcome of this study, more than 30 min of spontaneous circulation recovery, 30 h survival, and survival were performed after professional cardiopulmonary resuscitation to verify whether AVF affects the prognosis of cardiac arrest patients. Lastly, Kaplan–Meier curves, stratified into two groups with AVF devices and a normal manual defibrillator, were used to show the survival period, and the log-rank test was used to test the significant difference. Cox proportional risk regression was used to investigate the independent association between multivariable analytical gender and 80-day mortality. For each study subject, observation continued from the date of discharge to 30 days after or until death or censorship. The primary endpoint was death from all causes. Data were analyzed using SAS ver. 9.2 (SAS Institute Inc., Cary, NC, USA). If a *p*-value was less than 0.05, the finding was interpreted as statistically significant.

### 2.6. Equipment

**Figure 2:**
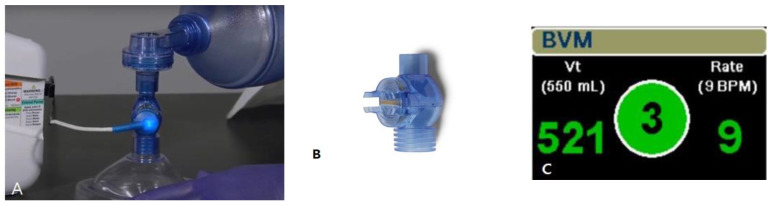
AccuVent™ flow sensor. (**A**) Accubent sensor with cable, (**B**) Accubent sensor, (**C**) audio ventilation feedback dashboard.

All devices were from the ZOLL X Series (ZOLL Medical Corporation, Chelmsford, MA, USA). AccuVent (ZOLL Medical Corporation, Chelmsford, MA, USA) is a disposable differential pressure-based flow sensor designed to measure the delivered ventilation rate and tidal volume and provide breath-by-breath feedback to the rescuer (Figure 2). The sensor is connected between the bag and the mask or endotracheal tube. The sensor is connected to a reusable cable, which is attached to the X Series defibrillator (ZOLL Medical Corporation, Chelmsford, MA, USA).

## 3. Results

### 3.1. Baseline Characteristics of the Study Population

Table 1 shows the general characteristics of the patients with out-of-hospital cardiac arrest collected. The overall average age of the patients with cardiac arrest outside the hospital was 70.36 ± 15.68, and 68 (56.1%) were male patients with cardiac arrest, which was more than the number of female patients (56.1% vs. 43.9%). Of the total OHCA patients, 13 (10.7%) cases had CPR performed by witnesses on site, and out of those 13, 7 (11.1%) were in the AVF patient group and 6 (10.3%) were in the NVF patient group. Of the total patients, a total of 51 (42.1%) patients applyed target temperature treatment (TTM). As a result of comparing the cases of ROSC within 30 min, there was a significant difference of 35 (55.5%) in the AVF-applied patient group and 26 (44.8%) in the NVF patient group (*p* = 0.031). When comparing the survival rate within 24 h, there was a significant difference of 31 (49.2%) in the patient group with AVF and 27 (46.5%) in the patient group with NVF (*p* = 0.001). In comparing the survival rate within 80 days between the two groups, there was no significant difference (33.3% vs. 22.4, *p* = 0.232).

### 3.2. Effect of AVF Device on the Prognosis of Patients with Out-of-Hospital Cardiac Arrest

The results showing the effect of the AVF device on the prognosis of CA patients are shown in Table 2 below. Patients applying AVF helped increase spontaneous circulatory recovery within 30 min and helped survival within 30 h (0.56 (0.37–1.85), *p* = 0.001 and 0.53 (0.31–1.93), *p* = 0.03). And it was found that the application of TTM had an effect on prognostic recovery (0.43 (0.21–0.73), *p* = 0.02). However, it did not affect the 80-day survival rate, good neurologic outcome (CPC 1–2), or survival to hospital discharge [7].

### 3.3. Survival Analysis

**Figure 3:**
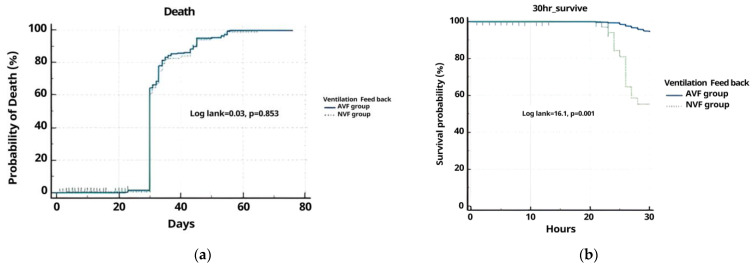
Kaplan–Meier death (**a**) and survival (**b**) estimates for the AVF group and NVF group in 80 days and 30 h.

Overall, 41.8% of cases survived for 80 days. We did not detect a difference in the probability of death within 80 days between the AVF group (57.3%) and the NVF group (42.7%). Figure 3 shows the Kaplan–Meier curve of survivors up to 80 days after CA. The log-rank test was not significant (*p* = 0.864). The calculated overall mortality rate was 58.1%. The NVF group mortality was higher in the AVF group. Overall, 50.9% of cases survived for 30 h. We did detect a significant difference in the 30 h survival in the AVF group (91.04%). Figure 2 shows the Kaplan–Meier curve of survivors up to 30 h after CA. The log-rank test was not significant (*p* = 0.0001) (Figure 3, Table 3).

As a result of investigating the effects of the AVF device and NVF device on mortality, it can be seen that neither group directly affects the mortality rate (HR: 0.9769 (0.4591–2.2825), *p* = 0.3180 vs. HR: 1.0237 (0.4381–2.1781), *p* = 0.0675) (Table 4).

## 4. Discussion

In this study, the use of audio ventilation feedback devices during professional cardiac resuscitation performed on patients with out-of-hospital cardiac arrest was related to the spontaneous circulation recovery rate within 30 min and survival rate within 30 h. It also helps increase the ROSC. However, it was found that it did not affect the 80-day probability of death and neurological prognosis. Studies that examined the relationship between survival rate and spontaneous recovery rates of ventilation with out-of-hospital cardiac arrest patients previously conducted showed that invasive positive ventilation during cardiopulmonary resuscitation limits lung loss and improves alveolar ventilation compared with ventilation using back valve masks [8,17]. In addition, the respiratory effects of 10/min and 20/min using mechanical ventilation during CPR and comparing the spontaneous circulation recovery rate, the spontaneous purification recovery rate was more than 50%, even though the gas exchange effect was high [18]. According to recent studies related to prognostic factors out-of-hospital cardiac arrest, it was found that age, a history of diabetes mellitus and renal failure, multivessel coronary artery disease on angiography, and time from pain onset to first medical contact were related to long-term outcomes [19,20,21,22,23,24]. And another study has revealed that brain oxygen supply can reduce mortality and appears as spontaneous circulation recovery and a good neurological prognosis [16,25,26]. This result showed that the recovery rate of spontaneous circulation increased despite providing more than twice the ventilation compared to the AHA guidelines in 2020. In fact, in the case of pediatric cardiac arrest patients, the 2020 AHA guidelines recommended increasing the ventilation rate to between 20 and 30 bpm, double the existing ventilation rate of 8 to 10 bpm [21]. However, in the case of adults, it has been strongly recommended to prevent hyperventilation in accordance with the guideline recommendations. The reason is that by increasing intrathoracic pressure, hyperventilation can reduce venous reflux and impair the hemodynamic efficacy of chest compressions and resuscitation [20]. Therefore, it has been found that preventing an increase in intrathoracic pressure during CPR increases the spontaneous circulation recovery rate. In this study, AVF showed that rescuers could receive feedback on ventilation speed and amount of ventilation, thereby preventing an increase in intrathoracic pressure, which increases the voluntary circulation recovery compared to the patient group without a feedback device. The reason why the feedback device can provide the correct ventilation speed and amount to the patient is that the main screen of the defibrillator provides a numerical value obtained via the combined sensor, and when the next ventilation is performed after one ventilation, the accurate ventilation rate can be determined. In addition, if the provider performs hyperventilation, a red alarm symbol is displayed and hyperventilation should thus be discontinued. Since such feedback can prevent an increase in intrathoracic pressure and a decrease in circulation, it can be interpreted that the spontaneous circulation recovery rate is higher than that of defibrillators without ventilation feedback. In the current study, it can be seen that the survival rate within 30 h increased in the patient group using the feedback device. These results prove that the appropriate ventilation power provided to cardiac arrest patients is absolutely helpful in restoring function by facilitating the oxygenation provided to the organs of cardiac arrest patients. Oxygen is essential for maintaining ATP regeneration and many other energy-consuming processes throughout the body that affect the oxidative phosphorylation and myocardial contraction function of mitochondria. In the event of cardiac arrest, ventilation can be temporarily omitted in the early stages of resuscitation efforts by prioritizing chest compression and defibrillation while utilizing oxygen available in the lungs, blood, and myocardium, but it is clear that spontaneous circulation recovery is impossible in a severely hypoxic environment. Therefore, efforts to secure an appropriate amount of oxygen are essential for the resuscitation of cardiac arrest patients.

This study had several limitations. First, data were collected from intubated patients. Use of the Accuvent device is not limited to intubated patients; it may be placed in the airway between the bag and the mask or the SGA. It is essential that the medical provider maintains a tight seal on the mask to ensure that the tidal volume measured by the device is delivered to the patient. However, the seal may be suboptimal where a facemask or SGA is deployed; this issue requires further study. Second, this study was conducted in a single region and a single institution, so it is difficult to generalize the results. Third, given the homogeneity of the careers and individual skills of each paramedic, it is difficult to generalize the possible performance of every paramedic. Fourth, the AVF group suffered OHCA more frequently in public areas, which is a potential modifier of outcomes. Finally, the AVF and NVF groups were not homogenous. Additional research will be needed to address these limitations.

## 5. Conclusions

An AVF device provides accurate ventilation and helps prevent an increase in intrathoracic pressure by preventing hyperventilation. Therefore, it helps to increase the 30 h survival rate and the ROSC rate within 30 min in patients with out-of-hospital cardiac arrest. However, AVF device use did not significantly affect the 80-day survival rate.

## Figures and Tables

**Table 1:** Baseline characteristics of the study population (N = 121).

Characteristics	Total (N = 121)	AVF Group (N = 63)	NVF Group (N = 58)	*p*-Value
Age (years)	70.36 ± 15.68	68.87 ± 15.01	70.98 ± 15.94	0.213
Gender				
Male	68 (56.1)	37 (58.3)	31 (53.4)	0.051
Female	53 (43.9)	24 (41.7)	27 (46.6)	0.111
Arrest location				0.101
Public area	73 (60.3)	43 (68.2)	30 (51.7)	0.012
Non-public area	48 (39.7)	20 (31.8)	28 (48.3)	0.042
Apply TTM	51 (42.1)	29 (46.0)	22 (37.9)	0.123
Time to CPR by bystander (min)	9.4 ± 3.43	6.5 ± 2.11	6.9 ± 2.88	0.981
CPR duration (min)	34.1 ± 11.23	39.4 ± 12.89	37.2 ± 10.21	0.126
Time from collapse to visit ED				
Witness collapse, yes	31 (25.6)	17 (26.9)	14 (24.1)	0.323
Bystander CPR, yes	13 (10.7)	7 (11.1)	6 (10.3)	0.332
ROSC within 30 min	69 (57.0)	43 (68.2)	26 (44.8))	0.031
ROSC (%) yes	58 (47.9)	35 (55.5)	21 (36.2)	0.041
Outcome				
Survival in 80 days	49 (40.4)	21 (33.3)	13 (22.4)	0.232
Survival at 30 h	59 (48.7)	31 (49.2)	27 (46.5)	0.001
Survival discharge	12 (9.9)	6 (4.9)	5 (8.6)	0.543
Death	67 (55.3)	31 (49.2)	34 (58.6)	0.654
Survival good outcome (CPC 1–2)	13 (10.7)	7 (11.1)	6 (10.3)	0.766

Values are presented as frequency (%) or mean ± standard deviation. TTM, target temperature treatment; ED, Emergengency department; CPR, cardiopulmonary resuscitation; ROSC, return of spontaneous circulation; CPC, cerebral performance category.

**Table 2:** Logistic regression for outcomes after use of AVF.

Outcome	Adjust OR	
	OR (95% CI)	*p*
ROSC within 30 min	0.56 (0.37–1.85)	0.001
Survival in 80 days	0.66 (0.25–0.87)	0.82
Survival at 30 h	0.53 (0.31–1.93)	0.03
Survival to hospital discharge	0.84 (0.39–0.83)	0.67
Good neurologic outcome (CPC 1–2)	0.68 (0.28–0.91)	0.73

OR, odds ratio; CI, confidence interval; ROSC, return of spontaneous circulation; CPC, cerebral performance category.

**Table 3:** Cox proportional hazard regression for survival between AVF and NVF groups.

Covariate	b	SE	Wald	OR (95% CI)	*p*
AVF group	0.2121	0.0822	2.1021	0.7731–1.2439	0.0380
NVF group	0.3563	0.1952	3.3311	0.9740–2.0937	0.0675

Harrell’s C-index = 0.503, 95% CI (0.400–0.605); OR, Odds ratio; AVF, audio ventilation feedback; NVF, non-ventilation feedback; HR; hazard ratio; CI, confidence interval.

**Table 4:** Hazard ratios (95% CI) for mortality in the AVF and NVF groups.

VF	HR	95% CI	*p*
AVF group	0.9769	0.4591–2.2825	0.3180
NVF group	1.0237	0.4381–2.1781	0.0675

VF, ventilation feedback; AVF, audio ventilation defibrillator; NVF, non-ventilation feedback defibrillator; HR, hazard ratio; CI, confidence interval.

## Data Availability

The data that support the findings of this study are available from the corresponding author (J.G.J.) upon reasonable request.
